# The Current Role of Parathyroid Fine-Needle Biopsy (P-FNAB) with iPTH-Washout Concentration (iPTH-WC) in Primary Hyperparathyroidism: A Single Center Experience and Literature Review

**DOI:** 10.3390/biomedicines10010123

**Published:** 2022-01-06

**Authors:** Łukasz Obołończyk, Izabela Karwacka, Piotr Wiśniewski, Krzysztof Sworczak, Tomasz Osęka

**Affiliations:** 1 Department of Endocrinology and Internal Medicine, Medical University of Gdańsk, 80-210 Gdansk, Poland; przepona@wp.pl (Ł.O.); piotr.wisniewski@gumed.edu.pl (P.W.); krzysztof.sworczak@gumed.edu.pl (K.S.); 2 Independent Public Healthcare Center of the Ministry of Interior and Administration in Gdańsk, Department of Surgery, 80-104 Gdansk, Poland; chirurgia@zozmswia.gda.pl

**Keywords:** parathyroid hormone washout concentration, primary hyperparathyroidism, parathyroid adenoma, hypercalcemia

## Abstract

Introduction. Primary hyperparathyroidism (PHPT) is a condition characterized by disorders of calcium–phosphate metabolism and bone metabolism caused by pathological overproduction of parathyroid hormone (PTH). The diagnosis of overt PHPT is based on the presence of clinical symptoms and laboratory abnormalities typical of this condition: hypercalcemia, hypercalciuria and elevated iPTH levels. Imaging studies are not used for diagnostic purposes; they are performed to localize the parathyroid glands prior to potential surgical treatment. Technetium 99 m sestamibi scintigraphy (Tc99 m-MIBI) is the gold standard in the assessment of pathologically altered parathyroid glands. Other diagnostic options include cervical ultrasound (US), computed tomography (CT), magnetic resonance imaging (MRI) and positron emission tomography (PET). Parathyroid biopsy (P-FNAB) with iPTH washout concentration (iPTH-WC) assessment is still an underestimated method of preoperative parathyroid gland localization. Few studies have reported the utility of US-guided P-FNAB in preoperative assessment of parathyroid lesions. The aim of the study was to present our experience with 143 P-FNAB with iPTH-WC assessment. Material and methods. Laboratory results, US findings, P-FNAB complications and comparison with other imaging techniques were described and analyzed. Results. In 133 (93.0) patients, iPTH washout-to-serum ratio exceeded threshold level 0.5 and were classified as positive results. Median iPTH-WC in this group was 16,856 pg/mL, and the iPTH-WC to serum iPTH ratio was 158. There was no correlation between iPTH-WC and serum PTH, serum calcium, parathyroid gland volume and shape index. In the group of 46 operated patients, 44 demonstrated positive iPTH-WC results, which corresponds to a sensitivity of 95.6%. In Tc99-MIBI, radiotracer retention was found in 17 cases (in 24 MIBI performed), which corresponds to a sensitivity of 52.2%. P-FNAB did not cause any major side effects −92.5% of all patients had no or mild adverse events after this procedure. Conclusions. P-FNAB with iPTH-WC is a reliable method in parathyroid adenoma localization during PHPT. Its sensitivity for diagnosis of PHPT is much higher than that of Tc99-MIBI, and in some situations, P-FNAB with iPTH-WC may even replace that method. Furthermore, cost-effectiveness of iPTH-WC is at least similar to that of Tc99-MIBI. Complications of P-FNAB are mild and we can describe this method as a safe procedure.

## 1. Introduction

Primary hyperparathyroidism (PHPT) is a condition characterized by disorders of calcium–phosphate metabolism and bone metabolism caused by pathological overproduction of parathyroid hormone (PTH) [1,2]. The classic symptomatology of PHPT includes symptoms related to hypercalcemia, bone changes and renal dysfunction in the form of nephrolithiasis [3,4]. The diagnosis of overt PHPT is based on the presence of clinical symptoms and laboratory abnormalities typical of this condition: hypercalcemia, hypercalciuria and elevated iPTH level [5,6,7]. Imaging studies are not used for diagnostic purposes; they are performed to localize the parathyroid glands prior to potential surgical treatment. Technetium 99 m sestamibi scintigraphy (Tc99 m-MIBI) is the gold standard in the assessment of pathologically altered parathyroid glands. Other diagnostic options include cervical ultrasound (US), computed tomography (CT), magnetic resonance imaging (MRI) and positron emission tomography (PET). Bilateral intraoperative neck exploration to identify and remove the hyper-functioning parathyroid tissue is the gold standard for the treatment of PHPT. However, the surgical approach of choice is minimally invasive parathyroidectomy (MI ptx) since the precise localization of the parathyroid lesion has become possible by imaging [8]. Preoperative localization of the parathyroid lesion is critical to identify suitable candidates for MI ptx, which results in a reduction of the invasiveness of the surgical technique [3].

Cervical ultrasonography (US) and Tc99 m-MIBI represent the first steps in parathyroid adenoma (PA) localization, with many centers using both methods in combination. This approach is very sensitive (90%) and highly accurate (97.2%) in PHPT, with greater experience resulting in more reliable results. Larger PAs (>1.8 cm) and higher preoperative ionized calcium levels (>1.49 mmol/L) are more reliably identified by sestamibi scan [9]. The imaging reveals 70–80% of PAs and also allows for ectopic parathyroid tissue to be localized. Tc99 m-MIBI, particularly when complemented by single emission computed tomography (Tc99 m-MIBI-SPECT), is a widely used imaging technique for the preoperative localization of PAs [10]. However, the test is also known to have lower sensitivity for small adenomas weighing 500 mg or less and in the presence of multiple gland pathology and persistent PHPT [9].

The diagnostic performance of US and Tc99 m-MIBI seems to be similar [11]. US may be useful as a supplemental study to confirm the localization of a parathyroid adenoma identified by Tc99 m-MIBI [12]. High-resolution US is safe, rapid and easy to perform by skilled hands. The sensitivity of US is 76–87% with a positive predictive value of 93–97% and a diagnostic accuracy of 88% [10]. By ultrasound, a PA is usually seen as a round or oval well-defined, hypoechogenic structure delineated by an echogenic line and contrasting with the overlying hyperechogenic thyroid tissue. Calcifications and cysts may be found in larger parathyroid adenomas [13]. Ultrasound is most useful in identifying parathyroid adenomas close to the thyroid gland or the upper cervical portion of the thymus. US provides a safe and quick imaging modality free of ionizing radiation; however, the imagining is operator-dependent and concomitant thyroid nodules may cause false-negative results [11]. Additionally, US is less useful in visualizing parathyroid pathology within the mediastinum or in retroesophageal or retropharyngeal location [12].

The ultrasound-guided parathyroid fine-needle aspiration biopsy (P-FNAB) is a minimally invasive, safe and effective method for the diagnosis of parathyroid lesions. The procedure can be performed to localize a parathyroid tissue and distinguish it from surrounding structures. The use of preoperative P-FNAB can significantly increase the accuracy of parathyroid gland (PG) localization [14]. However, the distinction of the different parathyroid lesions, including hyperplasia, adenoma and carcinoma, cannot be made solely on cytology [15]. Measurement of iPTH in P-FNAB washout fluids (intact parathormone washout concentration; iPTH-WC) complements cytology for identification of parathyroid tissue [16]. The procedure with iPTH washout had a superior performance compared with parathyroid scanning or US alone [14]. The preoperative P-FNAB may be also useful for localizing suspected parathyroid tissue in patients in whom previous exploratory parathyroidectomy (ptx) was unsuccessful, to determine ectopic locations of parathyroid tissue, particularly when the sonographic findings are not conclusive [17]. The intraoperative quantification of iPTH levels in the fluids of various tissues may also be used for identification of PGs during thyroidectomy and prevention of post-operative hypoparathyroidism [18]. The major limitation of P-FNAB with iPTH-WC is the need for initial identification of a potential parathyroid adenoma by ultrasonography. Moreover, the challenge in interpreting a study is the number of false-negative results. The potential risk of P-FNAB includes disruption of the lesion and seeding along the needle tract, causing separate secondary parathyroid lesions, called parathyromatosis [19].

Few studies have reported the utility of US-guided P-FNAB in preoperative assessment of parathyroid lesions. The aim of the study was to present our experience with 143 P-FNAB with iPTH-WC assessment, the safety assessment of FNAB of the parathyroid glands with iPTH-WC and an attempt to determine the cut-off value of iPTH-WC. Laboratory results, US findings, P-FNAB complications and comparison with other imaging techniques are described and analyzed.

## 2. Materials and Methods

### 2.1. Study Group

The study was conducted from 2017 to 2021 in the Department of Endocrinology and Internal Medicine in Gdansk. The study group consisted of 179 consecutive patients with suspicion of primary hyperparathyroidism PHPT who underwent P-FNAB with iPTH-WC assessment. Finally, examination results of 143 people with confirmed PHPT were included in the statistical analysis.

### 2.2. Inclusion Criteria

1. Confirmed PHPT:
  - documented PTH-related hypercalcemia (laboratory assessment at the same time) with:
  - serum iPTH >69 pg/mL (laboratory reference ranges 11–69 pg/mL)
  - serum total calcium >10 mg% (laboratory reference ranges 8.9–10.0 mg/dL)
  - additionally, if serum total calcium was between 10.0 and 10.99 mg/dL—at least two independent assessments on different occasions had to be documented
2. Age above 18 years old.

### 2.3. Exclusion Criteria

1. Patients with suspicion of enlarged parathyroid gland(s) but without laboratory abnormalities typical of PHPT.
2. Secondary or tertiary hyperparathyroidism.
3. Age below 18 years old.

### 2.4. P-FNAB Procedure

Before P-FNAB, peripheral blood for laboratory analysis (including iPTH and total calcium levels) was obtained. These results were included in the statistical analysis. The next step was to perform an ultrasound with parathyroid localization. If the parathyroid gland was detected, P-FNAB was performed. The consecutive stages of FNAF included:

- Disinfection of the operating field;
- Biopsy of suspected structure (freehand technique) with a standard needles 25 G (0.5 × 25 mm or 0.5 × 40 mm) or 23 G (0.6 × 25 mm or 0.6 × 40 mm);
- Visualization of the tip of the needle in PG according to authors’ own scale, QuOBo (Quality of Biopsy; see below for details);
- Aspiration of material—usually small amount in the tip of the syringe;
- Topping up with 0.9% saline up to approximately 1 mL (if necessary);
- Transfer of the material (iPTH washout) to a test tube;
- Immediate transfer to the laboratory and iPTH-WC assessment;
- Simultaneous compression of biopsied field;
- USG control directly after P-FNAB and 15–30 min later.

### 2.5. Laboratory Tests

Serum calcium concentration was determined by spectrophotometry using test kits from Abbott Laboratories. The coefficient of variation (CV) for intra-assay precision was <1.1%. iPTH concentration was determined by immunochemical method using the IMMULITE intact PTH kit by SIEMENS. The coefficient of variation (CV) for intra-assay precision was <5.7% (for mean iPTH range 72–662 pg/mL). No CV for higher concentrations of iPTH were mentioned. High-Dose Hook Effect was not observed up to 500,000 pg/mL. Usually iPTH-WC above 250,000–300,000 pg/mL were no further diluted. The highest iPTH-WC was 1,437,200 pg/mL. Positive results for iPTH-WC were considered when iPTH washout-to-serum ratio exceeded 0.5. This cut-off value was determined arbitrarily from the literature data and taking into account the unique methodology of sample dilution.

### 2.6. Ultrasonography

Ultrasound examinations were performed by a sonographer with many years of experience in examining the thyroid and parathyroid gland, using the GE Loqiq 7 SE machine, with a linear 8–15 MHz probe. The examination was performed without any preparation of the patient, in the supine position, with the head tilted back. This is the position where the thyroid and parathyroids are best accessible. In order to achieve proper contact with the skin surface, a layer of standard ultrasound gel was applied to the test site.

### 2.7. Parathyroid Quality of Biopsy Scale (QuOBo)

**Figure 1:**
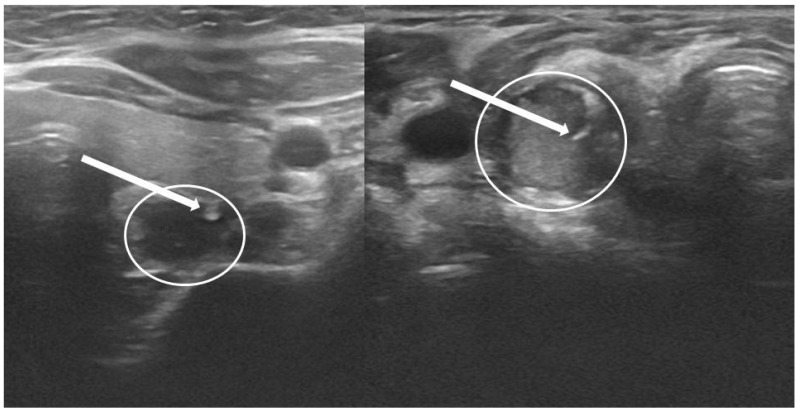
QuOBo score examples. Left image—QuOBo2; the tip of the needle (end of arrow) inside the parathyroid (in a circle) close to the border. Right image—QuOBo3; the tip of the needle (end of arrow) in the middle of the parathyroid (in a circle).

This scale, developed by the authors themselves, determines visibility of the needle tip in a suspected parathyroid gland. The scale ranges from 0 (worst visualization) to 3 (best visualization)—see details in Table 1 and examples in Figure 1. This scale was created in 2020, thus it was used only in part of the described biopsies—not included in statistical analysis.

### 2.8. Safety Protocol Scale and Compliance Scale

Scales were created in February 2021 and have been regularly used since with both ranges from 0 to 3. Details of all scales are in Table 1.

### 2.9. Statistical Methods

Descriptive statistics were calculated for demographic characteristics and clinical features. Continuous variables were described as medians and 1st–3rd quartiles (Q1–Q3). Categorical variables were analyzed using the two-sided Fisher’s exact test and continuous variables using the two-sample Wilcoxon rank-sum (Mann–Whitney) test. The study design allowed for investigation of diagnostic sensitivity of iPTH washout-to-serum ratio for confirmation of parathyroid tissue. An arbitrary cut-off value of 0.5 was chosen. Confidence limits for the sensitivity estimate were calculated based upon 10,000 resamples of the data set. A bootstrap method was used due to low number of false-negative results in the study sample [https://doi.org/10.1002/(SICI)1097-0258(20000215)19:3%3C313::AID-SIM370%3E3.0.CO;2-K (27 December 2021)]. *p*-values of less than 0.05 were considered statistically significant. All analyses were performed using R (version 4.0.5) and R Studio (version 1.4.1103) software.

## 3. Results

### 3.1. Study Group

A total of 143 patients with PHPT who underwent P-FNAB with iPTH-WC assessment were included in the analysis. The median age was 61 years (interquartile range, Q1–Q3, 50–69 years); 127 (89%) of the patients were women and 16 (11%) were men. The median serum calcium concentration was 10.8 mg/dL (Q1–Q3 10.4–11.5 mg/dL), phosphate 2.6 mg/dL (Q1–Q3 2.2–2.8 mg/dL) and iPTH 116 pg/mL (Q1–Q3 84–167 pg/mL). US revealed a suspicious lesion of the left lower PG in 43 (30%), the left upper PG in 25 (18%), the right lower PG in 58 (41%) and the right upper in 14 (9.9%). In two cases (1.4%), the location of suspicious PGs was in the upper mediastinum. The suspicious PGs on ultrasound presented the largest dimension of 1.54 cm (median, Q1–Q3 1.14–2.01 cm); their median volume was 0.52 mL (Q1–Q3 0.3–1.17 mL) and median shape index (ratio of longest to shortest dimension) was 0.49 (Q1–Q3 0.4–0.6). Seventy-four (51.7%) patients underwent MIBI parathyroid scan. In 44 of these 74 patients (59.5%), the radiotracer retention site coincided with suspicious PG on ultrasound. There was no difference in laboratory and ultrasound characteristics between men and women or between younger and older patients.

The median iPTH-WC was 13,373 pg/mL (Q1–Q3 2434–68,862 pg/mL) and was several orders of magnitude higher than concurrent serum iPTH concentration with the median ratio of 108 (Q1–Q3 27–593). There was no correlation between iPTH-WC and serum PTH, serum calcium, PG volume and shape index.

### 3.2. Study Group—Positive vs. Negative Results

In 133 (93.0) patients, iPTH washout-to-serum ratio exceeded threshold level 0.5 and were classified as positive results. With the exception of iPTH-WC, there was no difference in demographic, laboratory and ultrasound characteristics between patients with positive and negative results (see Table 2 for details).

Forty-six patients underwent surgical treatment. Adenoma was diagnosed in 42 (91.3%), hyperplasia in 3 (6.5%) and carcinoma in 1 (2.2%) patient. Of the operated patients, 44 had positive iPTH-WC results which corresponds to a sensitivity of 95.6%. Twenty-four (52.2%) operated patients had MIBI scans performed. The radiotracer retention was found in 17 cases, which corresponds to a sensitivity of 52.2%. The bootstrap-derived 95% confidence intervals for these estimates were 89.1–100% for iPTH-WC and 52.0–88.2% for MIBI scans.

Post-FNAB PG volume measurements were available for 37 consecutive patients. The volume of PGs increased and the median volume after FNAB was 95% greater than the median volume before FNA. Median volume and Q1–Q3 before and after P-FNAB was 0.44 (0.30; 0.90) and 0.86 mL (0.34; 1.56), respectively.

Measures of patients’ compliance, quality of P-FNAB performance (QuoBo) and P-FNAB-related adverse effects were available only for a subset of patients. The results are shown in Table 3. A very good compliance was noted in 85% percent of patients, the tip of the needle was seen inside the lesion in 94% of patients and 92.5% had no or mild adverse events.

## 4. Discussion

### 4.1. Embryology and Anatomy of PGs in Aspect of P-FNAB

**Figure 2:**
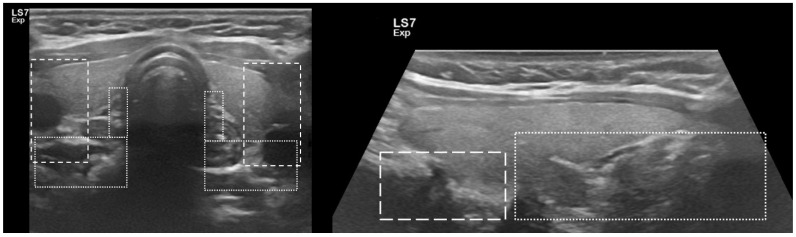
Area of the most common localization of parathyroid glands; superimposed on USG images. **Left part**—horizontal section; **right part**—longitudinal section. Superior parathyroid glands—lines; inferior parathyroid glands—dots.

Embryology of PGs is complicated. The superior PGs are derived from the fourth branchial pouch and are closely related to thyroid lobes. They have a short road of embryological migration and their final localization is stable on the posterolateral surface of the middle to superior thyroid lobe. The inferior PGs are derived from the third branchial pouch. They have a long migration road and probability of ectopic localization is more likely. Typically, they are situated in posterolateral surface of the lower part of thyroid lobe and ectopically in mediastinum and thymus [20]. Localization of the parathyroid is visualized in Figure 2.

**Figure 3:**
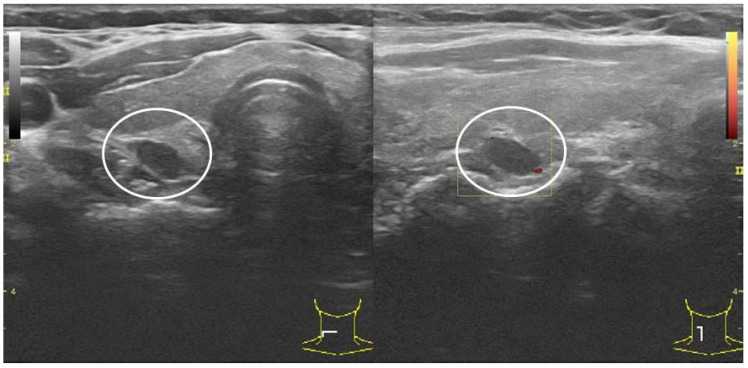
Small, slightly enlarged (5 × 8 × 8 mm) right superior parathyroid gland. No PHPT or iPTH-WC elevated.

**Figure 4:**
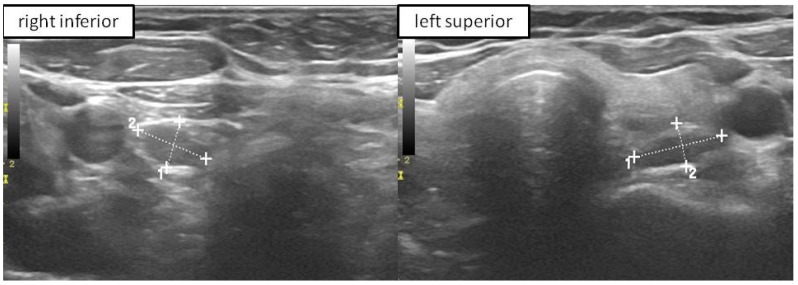
US of the patient with two enlarged PGs (confirmed with high iPTH-WC) i.e., right inferior and left superior (marked with measuring lines).

Parathyroids are small glands measuring approximately 2 × 4 × 5 mm. They have different shapes, but the oval (83%) and elongated (11%) shapes are the most common. Approximately 84% of population has typically four glands; this is crucial information from a practical point of view. Current ultrasound machines allow us to detect 2–3 mm structures, thus a healthy parathyroid is a potentially visible structure. Detecting a 2–5 mm parathyroid gland even with high levels of iPTH-WC does not confirm any parathyroid pathology (Figure 3). In our study, median shape index (shortest to longest diameter) was 0.49, confirming that usually PGs are oval. On the other hand, visualization of one enlarged parathyroid gland in patients with PHPT does not mean that there is only one pathological gland (Figure 4) [21].

Inferior PGs receive end-arterial blood from the inferior thyroid artery (ITA). Superior PGs receive blood from ITA in approximately 80–85% and only in 15–20% from the superior thyroid artery. Sometimes visualization of ITA may be helpful in PGs detection. Risk of ITA injury during P-FNAB is low.

The recurrent laryngeal nerve runs posteriorly to the inferior PGs, then crosses ITA and eventually passes the superior PGs running in front of it. In our material, no cases of recurrent laryngeal nerve or ITA injury were noted

### 4.2. P-FNAB

#### 4.2.1. Technical Approach to P-FNAB

From a technical point of view, PGs visualization and PGs biopsy are two opposite poles of the same procedure. Patients should know that P-FNAB is usually slightly longer than thyroid biopsy. Difficulty visualizing the needle in PGs and different volume of dilution buffer are probably the two most common reasons for wide ranges of PTH-WC values in our and other studies. Below are the most important patient/doctor factors and some of the authors’ tips to facilitate to the smooth and effective execution of P-FNAB (Table 4).

In our study, we performed biopsy in 143 patients with PHPT. Obviously, all biopsies were USG guided. Needle gauge were 23 or 25 and dilution buffer usually was 0.9% natrium chloride of approximately 1 mL. The number of needle passes ranged from one to three. This technique resembles other studies where needle gauge ranged from 21 to 27, the number of passes was usually one or two (maximum seven) and dilution buffer volume was from 0.5 to 1 mL of saline.

#### 4.2.2. Cut-Off Values

Despite similar P-FNAB technique, proposed cut-off values were different. The first proposed approach is a fixed cut-off value, which is the same for every patient. The other is patient specific, where cut-off value depends on serum iPTH levels. The most common suggested fixed values of iPTH-WC were between 40 and 103 pg/mL [22,23]. However, concentrations at even >1000 pg/mL were also proposed [14].

It must be noted that assessed iPTH-WC is diluted approximately 2–10 times with saline. In our material, a few patients had iPTH level above 400 pg/mL; therefore, fixed cut-off iPTH-WC value of 40 pg/mL might not provide answers, whether it was PA or just venous blood diluted by saline. Furthermore, in our material, patients with tertiary hyperparathyroidism (not included in the analysis) have iPTH even above 4000 pg/mL, thus the cut-off value of 40 pg/mL was only 1% of serum iPTH. That is why we kindly recommend adopting patient’s specific cut-off values. If iPTH-WC reaches similar concentration as serum iPTH, it means that “real”, not diluted, concentration is much higher. In our study (positive cases), iPTH-WC was much higher than iPTH, and the median iPTH-WC to serum iPTH ratio was 158. In our opinion, iPTH-WC equal to or higher than serum iPTH is a reasonable cut-off value as a proof that the visualized structure is an enlarged PG. Moreover, we suggest that lower iPTH-WC to serum iPTH ratio, i.e., between 0.5–1, should be interpreted as potential PG (see Table 5).

#### 4.2.3. Safety of P-FNAB

We developed a standardized security protocol in February 2021; therefore, it was used in part of the study group (40 cases). Nevertheless, no major complications were noted since the beginning of the study. According to the protocol, only three minor and three moderate complications were noted. Similar data were provided by Bancos et al. who described immediate complications in 5% of performed P-FNAB [14]. Castellana et al. did not find any major procedure-related complications in a large meta-analysis [24]. We had slightly more non-major complications, probably because of our standardized protocol with strict criteria (e.g., small skin hematomas were considered as minor complications) and double USG after P-FNAB procedure. We agree that P-FNAB is a safe procedure.

Another theoretical potential risk of FNAB biopsy is tumor seeding along the needle tract (parathyromatosis), as parathyroid tissue can adhere to and grow in various settings, which explains the high success rate with autotransplantations. FNAB may lead to disruption of the lesion and seeding along the needle tract, causing separate secondary parathyroid lesions [19]. Kendrick et al. did not note any case of parathyromatosis in 41 cases of P-FNAB [33]. On the other hand, parathyromatosis can be a complication of parathyroidectomy *per se*, thus sometimes the distinction as to what is the true cause of parathyromatosis may remain unresolved.

The complication during surgery may be caused by the dense fibrotic reaction that may occur following FNAB, leading to increased adhesions to surrounding structures, such as the recurrent laryngeal nerve and resulting in less clear tumor borders and increased operative time.

After P-FNAB, we observed that volume of biopsied PA was almost twice the previous size—from 0.44 mL before to 0.86 mL after the procedure. It might be related to small bleeding inside PA. Interestingly, this phenomenon might be a helpful suggestion that biopsied structure was indeed a PA. Such relationship has never been described before.

### 4.3. Other Imaging Techniques

#### 4.3.1. Tc99-MIBI

In our personal opinion, P-FNAB with iPTH-WC and Tc99 m-MIBI should not be considered as substitutes in every case. The main advantage of P-FNAB/iPTH-WC is its capability to detect small changes. Moreover, this examination has high specificity and positive predicted values [14]. On the other hand, there is a risk of misdiagnosing with iPTH-WC only. High iPTH-WC is not a piece of evidence that the biopsied PG is a cause of PHPT. We still could have missed ectopic, supernumerary PGs or it may be that the biopsied PG was just enlarged but not overactive. In our data, the sensitivity of the iPTC-WC in the correct preoperative localization of the pathologically changed PGs is statistically significantly higher than Tc99-MIBI (95.6% to 52.2%).

We suggest performing both of these procedures in young patients (risk of genetic syndromes e.g., MEN1, MEN2) with PHPT. Another group where iPTH-WC with Tc99-MIBI may be beneficial are patients with USG findings suggestive of many parathyroid-like lesions. In “classic” cases of PHPT, P-FNAB with iPTH-WC may replace Tc99-MIBI.

From a practical point of view, P-FNAB with iPTH is readily available, and crucial is the USG machine and the possibility of iPTH assessment. Unlike scintigraphy, no special workshop is necessary. In Poland, total costs (commercial price for the patient) of Tc99 m-MIBI and P-FNAB with iPTH-WC are almost the same.

#### 4.3.2. P-FNAB with Cytological Examination

Cytological examination of P-FNAB is difficult. Bancos et al. revealed than only 31% of P-FNAB were interpreted as parathyroid cells [14]. Although there is significant overlap in the cytomorphologic features of cells derived from parathyroid and thyroid gland, the presence of stippled nuclear chromatin, prominent vascular proliferation with attached epithelial cells and frequent occurrence of single cells/naked nuclei are useful clues that favor parathyroid origin [15]. Therefore, the main difficulty associated with FNAB of parathyroid neoplastic lesions lies in their differentiation and classification. Distinction of the different parathyroid lesions, including hyperplasia, adenoma and carcinoma, cannot be made solely on cytology [17,19]. As the histological presentations of both lesions are similar and parathyroid carcinoma is rare, this poses a diagnostic challenge. However, Caleo et al. assessed the possible cytological criteria to classify FNAB of parathyroid neoplasia [17]. Twenty-three FNAB samples of parathyroid neoplasia and parathyroid cysts were reviewed. The series included 18 parathyroid neoplasias, 4 cysts and 1 thyroid follicular neoplasm (histologically diagnosed as parathyroid adenoma). The preliminary observations suggested that evident nucleoli, mitoses and possibly a papillary-solid pattern may guide the differentiation between parathyroid adenoma and parathyroid carcinoma. Further observations on larger series are needed to confirm these findings.

#### 4.3.3. Multiphase CT

Multiphase CT is becoming a viable first-line imaging option, as it has equal to superior sensitivity for PG localization when compared with scintigraphy and US imaging [12]. Parathyroid four-dimensional computed tomography (4 DCT) may remain a problem-solving technique in challenging cases and after failed neck exploration. The use of tomographic imaging (SPECT and SPECT/CT) increases the detection rate of PHPT compared with planar 99 mTc-MIBI scintigraphy [11].

#### 4.3.4. PET Imaging

A prospective study on 100 patients with PHPT compared the accuracy of 18 F-fluorocholine PET/CT with that of 99 mTc-MIBI or 99 mTc-tetrofosmin SPECT/CT in the preoperative detection of parathyroid adenoma [34,35]. The 18 F-fluorocholine PET/CT was superior to the other methods studied, especially in the detection and localization of small parathyroid adenomas. 18-fluorocholine PET/CT proved more accurate than US or 99 mTc-sestamibi SPECT [36]. This method is more accurate than conventional morphological and functional imaging modalities (US or SPECT) for the detection of benign parathyroid lesions. It could, therefore, be a reliable tool in both primary and recurrent hyperparathyroidism [36].

#### 4.3.5. Parathyroid Venous Sampling (PVS)

PVS is performed in patients with persistent or recurrent disease after previous parathyroid surgery, when repeated noninvasive imaging studies are negative or discordant [37]. PVS, which may be followed by confirmatory parathyroid arteriography, if necessary, becomes important when the noninvasive imaging techniques are inconclusive (e.g., discordant functional and anatomical imaging) or fail to localize an adenoma [37].

## 5. Conclusions

P-FNAB with iPTH-WC is a reliable method in detection of PA during PHPT. Sensitivity during diagnosis of PHPT is much higher than Tc99-MIBI and in some situations may even replace that method. What is more, cost-effectiveness of iPTH-WC is at least similar to that of Tc99-MIBI. Complications of P-FNAB are mild and we can describe it as a safe procedure.

Main limitations of this method are various cut-off values for true positive results. Moreover, this method is sensitive for P-FNAB technique with saline dilution volume or numbers of passes during biopsy. Regardless of these limitations, iPTH-WC equal to or higher than iPTH seems to be a reasonable cut-off value for positive results. Furthermore, iPTH washout-to-serum ratio from 0.5 to 1.0 should be considered as most likely positive and such cases should be considered individually (e.g., another biopsy or Tc99 m-MIBI).

We recommend P-FNAB with iPTH-WC as an equivalent imaging method in PHPT.

## Figures and Tables

**Table 1:** Used scales during P-FNAB: QuOBo; Compliance and Complications scale description.

QuOBo Scale	Description
0	needle tip and the shadow of the needle is not visible—iPTH-WC should not be assessed
1	needle tip visible outside the parathyroid, but close to its borders
2	needle tip visible inside the parathyroid close to its borders
3	needle tip clearly visible in the middle of the parathyroid
**Compliance Scale**	**Description**
1 (weak)	e.g., strong hyperventilation, involuntary swallowing—weak visualization
2 (medium)	e.g., mild hyperventilation—the parathyroid visible during US, but not visible all the time during P-FNAB
3 (good)	perfect compliance—the parathyroid visible during whole procedure
**Complications Scale**	**Description**
0 (no complication)	no complications
1 (mild)	e.g., skin bruises of more 1 cm in diameter, pain in VAS scale 2–5
2 (moderate)	e.g., small hematoma visible in USG, pain in VAS > 5
3 (major)	e.g., necessary surgical intervention after FNAB, PG abscess, large hematoma visible in USG, hoarseness

**Table 2:** Comparison of positive and negative results of iPTH-WC measurement.

	iPTH-WC Diagnostic Category		
Characteristic	Negative, N = 10 ^1^	Positive, N = 133 ^1^	*p*-Value ^2^
Age	62 (54; 72)	60 (50; 69)	0.4
Male sex	2 (20%)	14 (11%)	0.3
Serum Ca [mg/dL]	11.10 (10.85; 11.70)	10.80 (10.40; 11.50)	0.13
Serum Pi [mg/dL]	2.70 (2.50; 2.90)	2.50 (2.20; 2.77)	0.2
Serum iPTH [pg/mL]	114 (90; 133)	116 (81; 168)	0.9
Largest dimension [cm]	1.79 (1.40; 2.00)	1.51 (1.13; 2.01)	0.4
Volume [mL]	0.58 (0.45; 1.13)	0.51 (0.29; 1.15)	0.7
Shape Index ^3^	0.50 (0.35; 0.59)	0.49 (0.40; 0.60)	>0.9
MIBI retention ^4^	6 (86%)	38 (57%)	0.2
Washout iPTH [pg/mL]	8 (7; 22)	16856 (4030; 72,214)	<0.001
Washout: serum iPTH ratio	0 (0; 0)	158 (38; 667)	<0.001

^1^ Median (Q1–Q3); n (%). ^2^ Wilcoxon rank sum test; Fisher’s exact test. ^3^ Ratio of longest to shortest dimension. ^4^ performed in 74 cases.

**Table 3:** Qualitative measures of P-FNAB performance.

Characteristic	Pts Assessed	n (%)
Compliance Scale	40	
0		1 (2.5%)
1		2 (5.0%)
2		3 (7.5%)
3		34 (85%)
Parathyroid Quality of Biopsy Scale	92	
0		1 (1.1%)
1		5 (5.4%)
2		22 (24%)
3		64 (70%)
Safety Protocol Scale	40	
None		34 (85%)
Mild		3 (7.5%)
Moderate		3 (7.5%)
Major		0 (0%)

**Table 4:** Technical approach and tips and tricks necessary to perform successful P-FNAB.

Patient	Doctor
− compliance; especially avoiding (involuntary) stress-induced hyperventilation − size of the parathyroid gland—the shortest dimension determines P-FNAB difficulty	− appropriate needle length; standard 2.5 cm needle may be too short (see details in Materials and Methods section) − precise visualization of the tip of the needle during P-FNAB—optimally according to standardized scale − proper preparation of material (similar volume of dilution buffer in every biopsy) and rapid transfer to the laboratory
Tips and tricks	
− biopsy is stressful for the patient ... be nice to the patient, smile − never, never rush − do not press hard on the probe—you will avoid squeezing of PG and reducing its smallest dimension... − that is why you should not skimp on US gel − be sure where is the tip of the needle − after P-FNAB press the puncture site firmly with your hand—you will avoid bleeding complications	

**Table 5:** Characteristics of selected studies (adapted from Castellana [24] and modified).

Author	Numbers of Patients with iPTH-WC	Needle Gauge	Number of Passes	Buffer (0.9% NaCl)	Cut-Off
Maser; 2006 [22]	6	25	2 to 7	1 mL	>40 pg/mL
Li; 2017 [25]	11	21	-	-	>57 pg/mL
Marocci; 1998 [26]	12	22	2	0.5 mL of PTH free serum	>100 pg/mL
Kuzu; 2012 [27]	12	22	-	1 mL	>1 × iPTH
Aydin; 2019 [28]	20	25	-	0.5 mL	>1 × iPTH
Ince; 2018 [29]	21	22	-	1 mL	>2 × iPTH
Boi; 2013 [23]	27	22–25	1 or 2	0.5 mL	103 pg/mL
Gokcay; 2018 [30]	29	25	-	1 mL	>1 × iPTH
Ozderya; 2017 [31]	44	27	1 or 2	1 mL	>1 × iPTH
Pekkolay; 2019 [32]	49	22	-	1 mL	>1 × iPTH
Bancos; 2012 [14]	67 (tertiary HPT included)	25	6	0.5–1.5 mL	>1000 pg/mL or >1 × iPTH
Obołończyk; 2021	143	23 or 25	1 to 2	1 mL	0.5–1 × iPTH PG possible >1 × iPTH PG certain

## Data Availability

Not applicable.

## Abbreviations

P-FNAB	parathyroid fine needle biopsy
iPTH-WC	intact parathormone washout concentration
PHPT	primary hyperparathyroidism
PG(s)	parathyroid gland(s)
PA(s)	parathyroid adenoma(s)
MI ptx	minimally invasive parathyroidectomy
Ptx	parathyroidectomy
Tc99 m-MIBI	Technetium 99 m sestamibi scintigraphy
US	cervical ultrasound
CT	computed tomography
PET	positron emission tomography
MRI	magnetic resonance imaging
(i)PTH	(intact) parathyroid hormone
